# Home Treatment of Eyes

**Published:** 1878-07

**Authors:** 


### The Bistoury.

ELMIRA, N. Y., JULY, 1878.

The BISTOURY is published Quarterly, upon the 1st of April, July, October and January, at Fifty Cents a year in advance.

### HOME TREATMENT OF EYES.

Almost every case of diseased eyes that applies to the ophthalmic surgeon for treatment, has first gone through with a system of poulticing, blistering and the application of "alum curd," tea leaves or some patent nostrum, to the inflamed organ.

Poulticing seems to be a universal home remedy for all manner of inflamed eyes. No matter what the disease may be, a poultice of slippery elm, flaxseed, or bread and milk becomes the exact thing required, (in the judgment of the home prescriber and his meddling neighbors) to restore the inflamed and painful eyes to duty again.

We desire to caution our readers against this abominable practice. Scarcely a day passes that does not bring us some poor unfortunate who has had his eyes injured, and sometimes ruined by these senseless applications. When an eye is inflamed, it does not require a poultice! Nothing could be further from what is really needed. By poulticing, more blood is attracted to the already too congested vessels, so increasing the pain and defeating the very object intended.

"Alum curd," and patent eye-waters are often used to allay inflammation. The first can do no harm, nor any particular good, (when applied externally and not allowed to come in contact with the eye itself), but the use of "eye-waters," is highly dangerous. We desire earnestly to impress this fact upon the minds of all readers of the BISTOURY. If you could but see a few of the unfortunates who apply to us with ruined eyes from the use of such preparations, you would not be surprised at our continually cautioning you against their use. Eyes that are badly inflamed and very painful, shunning the light, with hot scalding tears escaping from between the lids when attempts are made to separate them, are apt to have ulcers upon the *cornea*, which receive deposits of lead from these patent nostrums, producing a yellowish white spot, which can never be erased.

When your eyes are inflamed, apply to them cloths, wet in cold water, changing frequently. A little laudanum may be placed in the water. If the eyes are not speedily better, lose no time in seeking proper advice; but never poultice!

